# The Pesticide Chlordecone Promotes Parkinsonism-like Neurodegeneration with Tau Lesions in Midbrain Cultures and *C. elegans* Worms

**DOI:** 10.3390/cells12091336

**Published:** 2023-05-07

**Authors:** Valeria Parrales-Macias, Patrick P. Michel, Aurore Tourville, Rita Raisman-Vozari, Stéphane Haïk, Stéphane Hunot, Nicolas Bizat, Annie Lannuzel

**Affiliations:** 1 Paris Brain Institute—ICM, Inserm, CNRS, Hôpital de la Pitié Salpêtrière, Sorbonne Université, 75013 Paris, France; valeria.parrales@hotmail.fr (V.P.-M.); patrick.michel@icm-institute.org (P.P.M.); aurore.tourville@icm-institute.org (A.T.); ritaraisman@gmail.com (R.R.-V.); stephane.haik@gmail.com (S.H.); stephane.hunot@icm-institute.org (S.H.); 2 Faculté de Pharmacie de Paris, Université de Paris Cité, 75006 Paris, France; 3 Centre Hospitalier Universitaire de la Guadeloupe, Service de Neurologie, Faculté de Médecine de l’Université des Antilles, Centre d’Investigation Clinique (CIC) 1424, 97159 Pointe-à-Pitre, France

**Keywords:** cell culture model, chlordecone, dopamine neurons, neurodegeneration, Parkinsonism, tauopathy

## Abstract

Chlordecone (CLD) is an organochlorine pesticide (OCP) that is currently banned but still contaminates ecosystems in the French Caribbean. Because OCPs are known to increase the risk of Parkinson’s disease (PD), we tested whether chronic low-level intoxication with CLD could reproduce certain key characteristics of Parkinsonism-like neurodegeneration. For that, we used culture systems of mouse midbrain dopamine (DA) neurons and glial cells, together with the nematode *C. elegans* as an in vivo model organism. We established that CLD kills cultured DA neurons in a concentration- and time-dependent manner while exerting no direct proinflammatory effects on glial cells. DA cell loss was not impacted by the degree of maturation of the culture. The use of fluorogenic probes revealed that CLD neurotoxicity was the consequence of oxidative stress-mediated insults and mitochondrial disturbances. In *C. elegans* worms, CLD exposure caused a progressive loss of DA neurons associated with locomotor deficits secondary to alterations in food perception. L-DOPA, a molecule used for PD treatment, corrected these deficits. Cholinergic and serotoninergic neuronal cells were also affected by CLD in *C. elegans*, although to a lesser extent than DA neurons. Noticeably, CLD also promoted the phosphorylation of the aggregation-prone protein tau (but not of α-synuclein) both in midbrain cell cultures and in a transgenic *C. elegans* strain expressing a human form of tau in neurons. In summary, our data suggest that CLD is more likely to promote atypical forms of Parkinsonism characterized by tau pathology than classical synucleinopathy-associated PD.

## 1. Introduction

Due to the high parasite pressure promoted by the tropical climate, various categories of pesticides have been used in the French Caribbean islands of Guadeloupe and Martinique. Among them, the organochloride pesticide (OCP) chlordecone (CLD) was extensively employed from 1972 until 1993 for the control of the banana weevil, a major pest of banana plantations, before being banned because of its reported toxicity [1]. Although currently forbidden, CLD is still found in waterways and soil and consequently in food products of plant and animal origin, including seafood products. This explains why CLD is detectable in blood samples from a large proportion of the population in the French Caribbean Islands of Guadeloupe and Martinique [1,2].

In Guadeloupe, exposure to CLD has been recognized as a potential cause of a high frequency of prostate cancer [1]. In addition, epidemiological studies have shown a possible negative impact of prenatal exposure to CLD on cognitive and motor development during infancy [3,4]. In the United States, workers involved in CLD production developed severe neurological symptoms, including tremor, suggesting that CLD intoxication may also result in neuronal dysfunction and possibly contribute to neurodegeneration [5,6]. When administered orally to mice, CLD appears to accumulate preferentially in specific brain areas, including the striatum and the medulla/pons, where it also exhibits a longer retention time [7,8]. Curiously, only a limited number of studies have addressed the question of the low-grade chronic neurotoxic effects of CLD with cumulative exposure. This is particularly surprising in view of the fact that exposure to OCPs represents a major risk factor for Parkinson’s disease (PD) [9,10,11,12], a disorder that is characterized primarily by the presence of intraneuronal protein inclusions, termed Lewy bodies, which are composed mainly of α-synuclein (αS), and by a loss of dopamine (DA) neurons in the substantia nigra (SN), which causes the progressive impairment of motor control.

In Guadeloupe and Martinique, there is an overrepresentation of atypical forms of degenerative Parkinsonism whose clinical manifestations are generally more severe than those of typical PD [13,14,15]. Caribbean patients with atypical Parkinsonism show motor symptoms resulting from lesions of the nigrostriatal dopaminergic system, that are either not improved or only partially improved by treatment with L-DOPA, and nonmotor neurological symptoms such as early cognitive alteration, hallucinations and dysautonomic symptoms [15,16], which are indicative of cerebral lesions that extend well beyond the nigrostriatal dopaminergic system. The neuropathological examination of brains from atypical Parkinsonian Caribbean patients showed a loss of SN DA neurons together with hyperphosphorylated tau lesions, predominantly in the midbrain but also in other areas of the brain [17], suggesting that these Parkinsonian syndromes are in fact tauopathies with Parkinsonism. On the basis of experimental and clinical studies, we previously suggested that Annonaceae plant products consumed as food or beverages contributed to this neurological syndrome [18,19,20] through their content of the acetogenin annonacin, which promotes mitochondrial poisoning by the inhibition of complex I, a prominent causal factor for Parkinsonian neurodegeneration [21]. Until now, however, the possibility that CLD could also contribute to Caribbean degenerative Parkinsonism has not been addressed.

Our present aim was therefore to better characterize the toxicological profile of CLD using in vitro and in vivo model systems relevant to studying degenerative Parkinsonism. More specifically, we evaluated the neurotoxic profile of CLD in cultures of midbrain DA neurons [22,23], and its proinflammatory potential in culture systems of brain glial cells. In addition, we used the nematode *Caenorhabditis elegans* (*C. elegans*) as an experimental model organism to study both PD neurodegeneration and behavioral deficits [24,25,26,27,28,29,30].

## 2. Material and Methods

### 2.1. Chlordecone Treatments

CLD (#N12291 or #45379; Sigma-Aldrich, L’Isles d’Abeau Chesnes, France) was dissolved in pure DMSO to obtain a 10 mM stock solution, which was then stored at −20 °C until further use. When needed, CLD was further diluted to 500 µM using either an astrocyte conditioned medium (ACM) or S-medium, i.e., the two media used for cell culture experiments and *C. elegans* studies, respectively.

### 2.2. In Vitro Studies

#### 2.2.1. Pharmacological Reagents for Cell Culture Experiments

The inhibitor of lipid peroxidation Trolox-C (#238813), the iron chelator deferoxamine (D9533), the NMDA glutamate receptor blocker MK-801 (M107), the anti-inflammatory drug dexamethasone (D4902), the antimitotic cytarabine (Ara-C; C6645) and the DA uptake inhibitor GBR12909 (D052) were all purchased from Sigma Aldrich (L’Isles d’Abeau Chesnes, France). The two fluorogenic dyes used to monitor oxidative stress and mitochondrial membrane potential, dihydrorhodamine-123 (DHR-123; #D23806) and tetramethylrhodamine methyl ester (TMRM; #T668), were both obtained from ThermoFisher Scientific (Courtaboeuf, France). The TLR2 agonist PAM 3CSK3 (#tlrl-pms) was purchased from Invivogen (San Diego, CA, USA). To promote seeded αS aggregation, we used commercially available fibril seeds of recombinant human αS (ab218819; Abcam, Cambridge, UK).

#### 2.2.2. Animal Experimental Procedures

Mice were housed, handled and cared for in strict accordance with the European Union Council Directives (2010/63/EU). Experimental procedures were approved by the Committee on the Ethics of Animal Experiments Charles Darwin No 5 (Ce/2017/005).

#### 2.2.3. Neuronal and Glial Cell Cultures

Midbrain neuronal cell cultures were produced using gestational day 13.5 embryos from Swiss mice (Janvier LABS; Le Genest St Isle, France) following previously reported technical procedures with some modifications [23]. After the sacrifice of pregnant mice by CO_2_ inhalation followed by cervical dislocation, embryos were collected from the two uterine horns, and the midbrains dissected under a Nikon SMZ800 stereomicroscope using Moria #7 microsurgery forceps (Antony, France). After the careful removal of the meninges, tissue pieces were collected in a 15 mL sterile polypropylene conical tube containing 2 mL of Leibovitz L15 culture medium (Sigma Aldrich). Then, to facilitate brain tissue dissociation, the tissue samples were incubated at 37 °C with 2 mL of a phenol red solution containing 0.05% trypsin and 0.02% EDTA (#25300; ThermoFisher Scientific, Coutaboeuf, France). After 20 min of incubation, the trypsin solution was neutralized by the addition of 2 mL of Dulbecco’s Modified Eagle Medium (DMEM; Thermo Fisher Scientific) supplemented with 10% FCS (Biowest LLC, Les Ulis, France). The tissue pieces were then recovered in 2 mL of L15 medium and dissociated by repeated pipetting (8–10 strokes) using a Gilson pipette fitted with a sterile polypropylene blue tip with no filter (StarLab France, Orsay, France). L15 medium was then added to bring the total volume to 8 mL, and the cells in the suspension were mixed by the double inversion of the test tube. Tissue pieces that remained undissociated and cellular debris were then allowed to settle for 30 min, and 6.5 mL of the resulting supernatant of dissociated cells in suspension was transferred to another sterile polypropylene conical tube, while the remaining pellet was subjected to another round of trituration. The supernatants were combined for centrifugation at 1300 rpm for 5 min at 4 °C, and the dissociated cells were gently resuspended in 2 mL of L15 medium before plating. The dissociated cells in suspension were seeded at a density of 40–60 × 10^3^ cells/cm^2^ onto either an Ibidi µ-slide 8-well glass bottom (CliniSciences, Nanterre, France) or Nunc 48-well multiwell plates (Roskilde, Denmark) precoated with 1 mg/mL of polyethylenimine (PEI; P3143; Sigma Aldrich) dissolved in a pH = 8.3 borate buffer [31].

The cultures were initially maintained in a Neurobasal-A medium (#10888022; Thermo Fisher Scientific) supplemented with a B27 supplement minus antioxidants (#10889038; Thermo Fisher Scientific), a N2 mix (#17502048; Thermo Fisher Scientific), a cocktail of penicillin/streptomycin, and 1% fetal calf serum (FCS; Biowest LLC). Two and eighteen hours after plating, Ara-C was added to the cultures at a concentration of 0.8 µM to halt glial cell proliferation. On day 4 in vitro (DIV), the plating medium was completely removed and replaced by ACM, prepared as described previously [23]. These cultures contained 2–3% tyrosine hydroxylase (TH)-positive neurons with a dopaminergic neurotransmitter phenotype [32].

Pure microglial cell cultures were obtained as previously described using a technique that relies on the preferential adhesion of microglial cells to PEI [31,33]. Briefly, brains from postnatal day 1 C57BL/6J mouse pups (Janvier LABS) were removed by dissection, and the meninges were stripped away, after which brain tissue samples were dissociated by repeated pipetting in L15 Leibovitz medium. After two rounds of trituration, the supernatant containing dissociated cells was centrifuged at 1000 rpm for 5 min at 4 °C. The resulting pellet was suspended in DMEM supplemented with 10% heat-inactivated FCS and 1% penicillin/streptomycin solution (defined as a complete medium). A cell suspension resulting from the trituration of two mouse brains was plated in PEI-coated Corning T-75 culture flasks (Sigma-Aldrich) containing the complete medium. The cultures were washed once with the complete medium after 2 days in vitro, and microglial cells were then maintained at 37 °C in a humidified atmosphere with 5% CO_2_ without any other culture medium change until the completion of microglial cell isolation, which was generally observed 14–16 days after plating under these conditions. The average yield ranged from 4–5 × 10^6^ CD45^+^ microglial cells/T-75 culture flask with this protocol.

The isolation of astrocytes was obtained through a procedure similar to that used for microglial cells with, however, the use of laminin (1 µg/mL; Sigma Aldrich) dissolved in distilled water as a coating. A DMEM/F-12 nutrient mixture (#21331; Thermo Fisher Scientific) supplemented with 10% FCS and 1% of an antibiotic cocktail was used as an isolation medium. In addition, the cultured cells were treated twice a week with clodronate liposomes (3 μg/mL; Liposoma BV, Netherlands) to eliminate residual microglial cells. A monolayer of glial fibrillary acidic protein (GFAP)^-^positive astrocytes was generally obtained after 12–14 days of cultivation.

After isolation, microglial cells and astrocytes were dislodged from the culture flasks by mild trypsinization (5–6 min) to produce subcultures. In both model systems, we used the TLR2 agonist PAM3CSK3 as a reference inflammogen [34].

#### 2.2.4. TNF-α Measurement

Tumor necrosis factor alpha (TNF-α) was quantified using an ELISA Kit (BMS607-3TEN, Thermo Fisher Scientific). Briefly, the culture medium used to maintain glial cells was recovered 24 h after the initiation of the test treatments and then frozen at −20 °C for subsequent analysis. TNF-α was measured according to the manufacturer’s instructions using aliquots (50 μL) of undiluted samples. The sample absorbance was read at 450 nm using a SpectraMax M4 spectrophotometer (Molecular Devices, Sunnyvale, CA, USA). ELISA standard curves were generated using a four-parameter logistic curve model (GraphPad Prism 8, GraphPad Software; San Diego, CA, USA).

#### 2.2.5. Immunodetection Protocols for Cell Culture

Cultures were fixed for 12 min at room temperature with 3.7% formaldehyde diluted in Dulbecco′s phosphate buffered saline (PBS) and then washed twice with PBS before incubation with primary antibodies for 24–48 h at 4 °C. The primary antibodies used for the cell culture protocols are listed in Table 1. Antibodies were pre-diluted in 0.2% Triton X-100, except for CD45, which was diluted in PBS only. We used anti-mouse/rabbit IgG (H+L) or anti-chicken IgY (H+L) conjugated to Alexa Fluor fluorescent dyes as secondary antibodies (Thermo Fisher Scientific).

#### 2.2.6. Cell Counting in Cell Cultures

For cell counting operations in culture, we used a Nikon TE 2000 inverted microscope (Champigny-sur-Marne, France) equipped with an ORCA-ER digital camera and the HCimage software (Hamamatsu Photonics, Massy, France). The number of TH^+^ neurons was estimated by visually inspecting 10–15 visual fields that were randomly selected for each treatment condition with a 10× objective. The percentages of TH^+^ somas with elevated p-Tau (AT8) immunostaining or with p-αS aggregates (p-αSa) were determined with a 20× objective by counting 10–15 visual fields randomly chosen in each culture well for each treatment condition [23].

#### 2.2.7. Tritiated-DA Uptake

We performed the uptake of tritiated DA to evaluate the functional integrity and synaptic function of DA neurons as described [22]. Briefly, the treatments were terminated by culture medium removal, and the cells were incubated at 37 °C in PBS-glucose (5 mM) containing 25 nM [3H]-DA (NET673; 40 Ci/mmol; PerkinElmer, Courtaboeuf, France). After 15 min, the PBS solution containing the excess of radiochemical was discarded, andcultured cells were washed twice with PBS glucose before a lysis step with 1% Triton-X 100 in distilled water. Cell lysates were finally recovered in 2 mL of Econofluor-2 (#6NE9699; PerkinElmer), and the radioactivity retained by DA neurons was quantified with a Tri-Carb 4910TR liquid scintillation spectrometer (PerkinElmer).

#### 2.2.8. Measurement of Reactive Oxygen Species (ROS) and Mitochondrial Membrane Potential (ΔΨm)

The ACM used for cell culture maintenance during treatments was removed and replaced by PBS supplemented with glucose (5 mM). Thereafter, the cultures were exposed sequentially to the mitoprobe TMRM (ab228569; Abcam, Cambridge, UK) at 50 nM and the ROS indicator DHR-123 (D23806; Thermo Fisher Scientific) at 25 µM [35,36]. After 30 min of incubation, the cultures were washed thoroughly to remove the excess fluorescent dyes and then reincubated in PBS glucose containing the same test treatments as before imaging. As reference treatments, we used H_2_O_2_ (250 µM) to stimulate ROS production and the uncoupler of oxidative phosphorylation carbonyl cyanide-p-trifluoromethoxyphenylhydrazone (FCCP; 1 µM) to stimulate mitochondrial depolarization. H_2_O_2_ and FCCP were added 2 h and 10 min before the addition of fluorogenic probes, respectively. Note that FCCP remained present during all steps of the imaging protocol. For each culture condition, fluorescent images from at least 5 randomly chosen fields were acquired with a 40x fluorescence objective using a Nikon TE 2000 inverted microscope equipped as described before. The excitation and emission wavelengths for DHR-123 were 490 nm and 525 nm, respectively. For TMRM, the corresponding values were 548 nm and 575 nm, respectively. The results are expressed as the percentage of change in fluorescence intensity relative to the baseline in control cultures. The open-source software *FIJI* [37] was used to quantify the fluorescent signals.

### 2.3. In Vivo Studies

#### 2.3.1. *C. elegans* Lines

We used the Bristol *N2* strain as a control line. As reference lines, we used loss-of-function mutant strains deficient in genes orthologous to TH (*cat-2*; *e1112*) [38] and the presynaptic DA transporter (DAT) (*dat-1*; *ok157*) [39]. To monitor neurodegenerative changes, we used transgenic lines expressing the GFP fluorescent reporter in dopaminergic neurons (control^GFP^; *dat-1p::gfp*; pNB30) [27], and in serotoninergic (*tph-1p::gfp*; *zdls13IV;* SK4013) and cholinergic (*unc-17p::gfp*; *vsIs48,* LX929) neuronal systems (*Caenorhabditis Genetics Center*, University of Minnesota, St. Paul, MN). We also used the tau transgenic line (*aex-3p::hTau*; CK144) with panneuronal expression of the human wild-type 1N4R tau isoform. This line was a generous gift from BC Kraemer (University of Washington, Seattle, WA 98104, USA) [40].

#### 2.3.2. Growth and Maintenance of *C. elegans* Strains

Worms were generally maintained in Petri dishes using a solid nematode growth medium (NGM) seeded with the OP50 strain of *E. coli* as previously described [41]. Synchronized worms were prepared with the standard bleach method [42]. Briefly, gravid adults were lysed with a bleaching solution (NaOH 500 mM; bleach 1%) to isolate the embryos, which were then hatched on a medium without food to prevent development and thereby obtain a synchronized population.

#### 2.3.3. *C. elegans* Liquid Culture and Treatments

Synchronized first larval stage (L1) animals were initially grown in a solid NGM Petri dish medium for 3 days at 20 °C without any test treatment to prevent potential interference with normal development. The dishes were then washed with an M9 medium, and the worms recovered by sedimentation were resuspended in an S-medium containing ampicillin (50 ng/mL) (A9393, Sigma Aldrich), nystatin (40 µM) (N6261, Sigma Aldrich) and the *E. coli* OP50 strain (*Caenorhabditis Genetics Center*; Univ Minnesota, Min, Saint Paul, MN, USA) as a food source, as previously described [42,43]. Nematodes in suspension were then transferred to 48-well multiwell plates at a final concentration of 1 worm/µL in the presence of the thymidylate synthase inhibitor 5-fluoro-2-deoxyuridine (#F0503; Sigma Aldrich) to prevent the hatching and growth of new worms. For the evaluation of test treatments, multiwell plates were inserted in a clear plastic box humidified with damp paper and maintained at 20 °C under continuous agitation (150 rpm).

#### 2.3.4. Quantification of the Survival of *C. elegans* Neuronal Cell Populations

Worms were fixed in PBS with 4% paraformaldehyde (PFA) before mounting on glass slides for visual inspection under a Leica DM2000 LED microscope equipped with a 60× oil immersion objective. The impact of treatments on neuronal survival was measured by visually quantifying the neuronal somas and, when specified, the dendrites of green fluorescent protein (GPF)-tagged populations of neuronal cells. Quantitative data were obtained by analyzing 30 worms per experimental condition, and the experiments were carried out in triplicate. Specifically, we quantified the number of dopaminergic somas in the head (CEP, ADE) and body (PDE). We also estimated the dendrite integrity from CEP neurons. NSM and ADF serotoninergic neurons and their dendrites were also counted in the heads of the worms. The numbers of cholinergic neurons were estimated in the ventral cords of the worms. Whole-animal images were acquired for living worms using an epifluorescence AZ100M Nikon macroscope. Images of neuronal cells and their dendrites were acquired using a Zeiss Apotome.2 imaging system (Zeiss, Rueil Malmaison, France) or an inverted Leica TCS SP8 confocal microscope (Leica, Nanterre, France) using a x63 oil immersion objective.

#### 2.3.5. Immunostaining of *C. elegans* Samples

After the termination of treatments, the worms were immunostained using a variation of the freeze-crack method from Albertson and colleagues [44]. Briefly, the worms were positioned onto Superfrost microscope slides (Fisher Scientific) previously coated with a drop of poly-L-lysine (Sigma-Aldrich) for 15 min at 60 °C. Then the worm bodies were sliced in half using a 25 G needle, and a coverslip was set on top of the worms before placing the slide on dry ice. The coverslip was removed to physically pull the cuticle from the nematodes, and fixation was performed with a solution of 4% PFA diluted in methanol. Then, the samples were washed once with PBS containing 1% Triton X-100 (PBS-T) and incubated overnight at 4 °C with 50 µL of the primary anti-total tau or anti-p-tau antibody (Table 1) diluted in a blocking solution. After washing with PBS-T, the worms were incubated in a solution containing the secondary antibody for 2 h at RT. The slides were mounted with 8 µL of anti-fade gold (Life Technologies) and stored at 4 °C until analysis.

#### 2.3.6. Quantification of Immunosignal Intensity

Image sections of the worm heads or bodies were acquired with a Leica TCS SP8 confocal microscope using a x63 oil immersion objective. Stacks of 30 images were acquired per worm using a 0.25 µm Z step size. The intensity of the p-tau immunosignal in neurons from the region of interest (ROI) was quantified using *FIJI* software [37].

#### 2.3.7. Behavioral Analysis

To evaluate the integrity and function of DA neurons, we used the basal slowing response (BSR) method [45]. Briefly, upon completion of the test treatments, the worms were washed with the M9 medium and transferred onto a solid medium supplemented with or without food (bacteria). Then, the crawling velocity was monitored by tracking the worms on short movies (1 min, 7 fps) acquired with an AZ100M Nikon microscope. The image analysis was performed with the Imaris imaging software from Oxford Instruments (Abingdon, Oxfordshire, UK). To evaluate the effects of L-DOPA, 5 mM of L-DOPA (Sigma-Aldrich) was added to *E. coli* seeded agar plates 3 h before testing. Quantitative data for the behavioral assays were derived from three independent experiments, each with 30 worms analyzed per experimental condition.

### 2.4. Statistical Analysis

Data are presented as the mean ± SEM. For the cell culture experiments, the data values were obtained from three independent sets of experiments. For the *C. elegans* experimental studies, each data point was derived from at least three independent experiments, except when noted. Data were generally analyzed using one-way analysis of variance followed by post hoc tests. For all comparisons against a single group, we used Dunnett’s test, and for all pairwise comparisons the Student–Newman–Keuls (SNK) or Tukey’s tests. The two-tailed unpaired *t*-test was used when comparing the mean values from two independent groups. The overall survival of *C. elegans* animals was illustrated with Kaplan–Meier curves and the log-rank (Mantel–Cox) test was used for statistical comparisons. All statistical analyses were performed with GraphPad Prism 9.0 software.

## 3. Results

### 3.1. CLD Promotes Concentration- and Time-Dependent Dopaminergic Cell Loss in Mouse Midbrain Cultures

**Figure 1:**
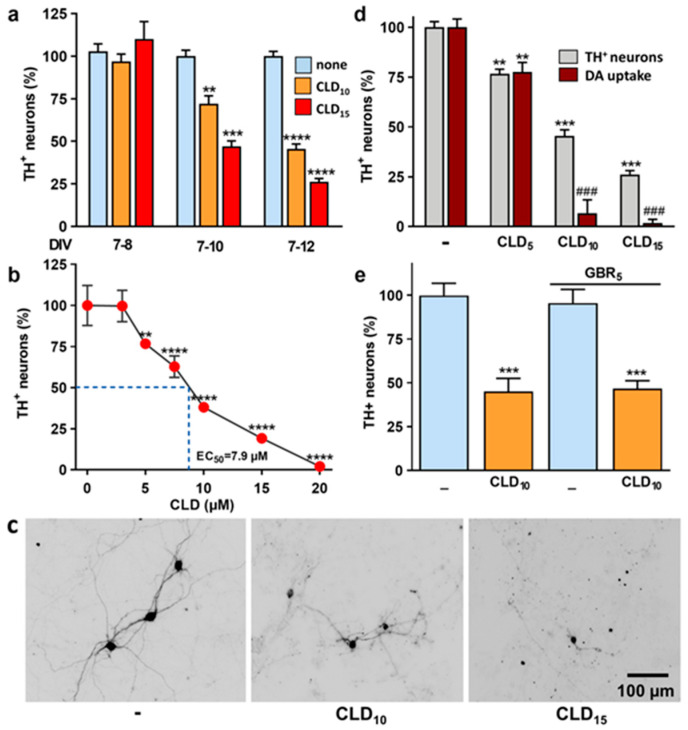
Characterization of CLD neurotoxic effects on midbrain-cultured DA neurons. (**a**) Counts of TH^+^ neurons in midbrain cultures exposed to 10 and 15 µM of CLD for 1, 3 and 5 days. Data are means ± SEMs (*n* = 6). ** *p* < 0.01, *** *p* < 0.001, **** *p* < 0.0001 vs. untreated corresponding controls. One-way ANOVA followed by Dunnett’s test. (**b**) Counts of TH^+^ neurons in midbrain cultures exposed to increasing concentrations of CLD (3–20 µM) for 5 consecutive days. Data are means ± SEMs (*n* = 6). ** *p* < 0.01, **** *p* < 0.0001 vs. untreated controls. One-way ANOVA followed by Dunnett’s test. (**c**) Illustration of the effects of a 5-day treatment with 10 and 15 µM of CLD on the number of TH^+^ cells and their morphology. (**d**) Comparison of the impact of CLD (5, 10, 15 µM) treatment on TH^+^ cell numbers and DA uptake. Data are means ± SEMs (*n* = 6). ** *p* < 0.01, *** *p* < 0.001 vs. corresponding controls. ^###^ *p* < 0.001 vs. TH^+^ cell numbers at the same concentration of CLD. One-way ANOVA followed by SNK test. (**e**) Impact of the DA uptake inhibitor GBR12909 (5 µM) on TH^+^ cell numbers in cultures treated or not treated with 10 µM of CLD. Data are means ± SEMs (*n* = 6). *** *p* < 0.001 vs. untreated control cultures. One-way ANOVA followed by Dunnett’s test.

We initially monitored the kinetics of CLD-induced DA cell death using DIV 7 midbrain cultures treated with the pesticide for different time periods. Specifically, we measured the survival of DA (TH^+^) neurons in midbrain cultures after 1, 3 or 5 days of exposure to 10 or 15 µM of CLD (Figure 1a). We established that DA neurons died progressively over the 5-day exposure to CLD. If TH^+^ cell loss was generally not detectable after 1 day of intoxication with either 10 or 15 µM of CLD, a significant number of TH^+^ cells had died after 3 days of exposure to both concentrations. DA cell loss was maximal 2 days later, i.e., after 5 days of CLD treatment (Figure 1a). Specifically, we established that 53% and 74% of TH^+^ neurons were killed after a 5-day exposure to 10 and 15 µM of CLD, respectively. Using a larger range of concentrations of CLD (3–15 µM) and a 5-day exposure time, we graphically estimated the effective concentration of CLD killing 50% of DA neurons (EC_50_) to be 7.9 µM (Figure 1b). Representative microphotographs of midbrain DA neurons exposed for 5 days to 10 and 15 µM of CLD are depicted in Figure 1c. For 10 µM of CLD, the TH^+^ cell somas remaining in the cultures generally had a shrunken appearance and presented a reduced neuritic network, suggesting that these neurons were already dysfunctional and irreversibly committed to death. Note that these effects were even more dramatic for 15 µM of CLD (Figure 1c).

To complement TH^+^ cell counting, we used tritiated-DA uptake as an index of DA cell survival and function [46]. Our results showed that the reduction in DA uptake was proportionally more important than the loss of DA neurons at both 10 and 15 µM of CLD (Figure 1d), confirming that DA cell death induced by CLD was preceded by neuronal dysfunction at both concentrations. However, no difference between TH^+^ cell counts and DA uptake was observed at 5 µM of CLD, a concentration that exerted only minimal toxic effects under the present conditions.

In addition, we showed that cotreatment of the cultures with the inhibitor of DA uptake GBR-12909 (5 μM) failed to protect against CLD-induced dopaminergic neurotoxicity, indicating that CLD does not accumulate in DA neurons via the high affinity transporter for DA (Figure 1e), a potential gateway for some dopaminergic toxins [47]. Consistent with this observation, CLD neurotoxic effects were not restricted to dopaminergic neurons, as neuronal cells labeled by the panneuronal marker MAP-2 were also affected by CLD within the same range of concentrations (not shown).

Note that the vulnerability of DA neurons to CLD was not increased when the pesticide was applied to older cultures, suggesting that neurodegeneration is not significantly impacted by the state of maturity of cultured neurons (Figure S1).

### 3.2. Neurodegeneration Induced by CLD Is Preceded by an Emission of ROS and a Drop in ΔΨm

**Figure 2:**
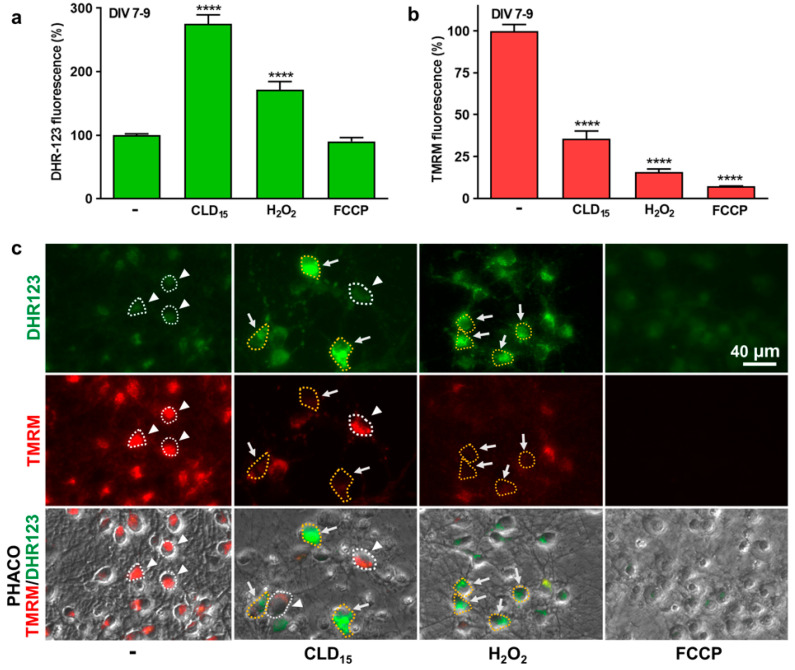
CLD-mediated ROS production correlates with a drop in ΔΨm in cultured midbrain neurons. (**a**) Midbrain cultures exposed or not exposed to 15 µM of CLD between DIV 7 and 9 and then processed for ROS measurement with the fluorescent probe DHR-123. ROS measurements were also performed in sister cultures acutely exposed to 250 µM of H_2_O_2_ or 1 µM of FCCP. Data are means ± SEMs (*n* = 3). **** *p* < 0.0001 vs. untreated control cultures. One-way ANOVA followed by Dunnett’s test. (**b**) Cultures exposed or not to 10 µM of CLD between DIV 7-9 and then processed for ΔΨm measurements using TMRM. Changes in mitochondrial membrane potential were also evaluated in sister cultures acutely exposed to 250 µM of H_2_O_2_ or 1 µM of FCCP. Data are means ± SEMs (*n* = 3). **** *p* < 0.0001 vs. untreated control cultures. One-way ANOVA followed by Dunnett’s test. (**c**) Concomitant changes in ROS production (green; upper panel) and ΔΨm (red; middle panel) within the same visual fields. The lower panel provides a combined illustration of the two fluorescent signals merged with the corresponding phase contrast (PHACO) image. White arrows point to some neuronal cells in which the elevation of ROS levels correlates with a drop in ΔΨm. Corresponding cell bodies are surrounded by a yellow dotted line. White arrowheads point to some neuronal cells in which ROS production remains low when ΔΨm is at control levels. Corresponding cell bodies are surrounded by a white dotted line.

Using the fluorogenic probe DHR-123, we showed that ROS were increased above threshold levels in a significant fraction of midbrain neurons 2 days following exposure to 15 µM of CLD (Figure 2a,b). Generally, ROS-producing neurons were identifiable by phase contrast illumination, as they exhibited swollen cell bodies. As expected, the DHR-123 fluorescent signal was also increased in neuronal cells in midbrain cultures exposed to 250 µM of H_2_O_2_ for 2 h. Neuronal ROS production in CLD- and H_2_O_2_-treated cultures was associated with a drop in ΔΨm (Figure 2b,c), visualized simultaneously in the same cultures through the reduction in the fluorescence signal emitted by TMRM, a cell-permeant dye that accumulates in active mitochondria with intact membrane potentials [48]. As expected, the TMRM fluorescent signal was also strongly reduced by transient treatment with 1 µM of FCCP, an uncoupler of mitochondrial oxidative phosphorylation that disrupts ΔΨm and ultimately induces cell death. ROS, however, were not significantly elevated under these conditions.

It is worth noting that treatments with the capacity to curtail oxidative stress-mediated insults [36,46] or to potentially stimulate aerobic glycolysis [18] were unable to protect DA neurons from degeneration (Figure S2).

### 3.3. CLD Stimulates p-tau but Not p-αS Expression in DA Neurons

**Figure 3:**
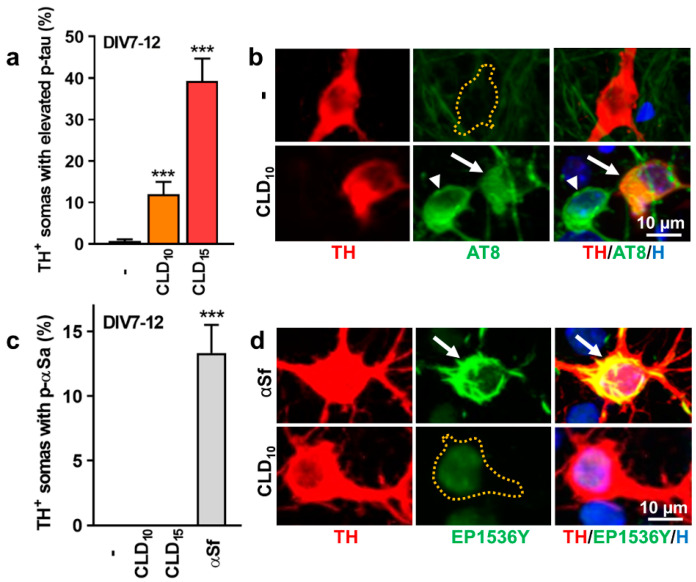
Impact of CLD exposure on p-tau or p-αS expression in midbrain DA neurons. (**a**) Percentage of TH^+^ somas with increased expression of p-tau (AT8 immunosignal) in midbrain cultures treated from DIV 7 to 12 with 10 or 15 µM of CLD. Data are means ± SEMs (*n* = 6). *** *p* < 0.001 vs. untreated control cultures. One-way ANOVA followed by Dunnett’s test. (**b**) Representative illustration showing an increase in the p-tau immunosignal in a TH^+^ neuron (white arrow) of a midbrain culture exposed between DIV 7 and 12 to 10 µM of CLD. Note that the AT8 immunosignal is also elevated in another neuron (white arrowhead) that does not express TH. (**c**) Percentage of TH^+^ somas with increased expression of p-αS (EP1536Y immunosignal) in midbrain cultures treated between DIV 7 and 12 with 10 or 15 µM of CLD. Positive controls were exposed to 7 µg/mL of αSf during the same time period. Data are means ± SEM (*n* = 6). *** *p* < 0.001 vs. untreated control cultures. One-way ANOVA followed by Dunnett’s test. (**d**) Representative illustration showing that the p-αS immunosignal is not increased in an individual TH^+^ neuron exposed to 10 µM of CLD between DIV 7 and 12, whereas a strong increase in the signal is observed in a TH^+^ neuron from a sister culture exposed to 7 µg/mL of αSf during the same time period. In (**b**,**d**), yellow dotted lines represent virtual boundaries of TH^+^ somas without p-tau or p-αS immunosignals, respectively.

We next evaluated the potential of CLD to stimulate the expression of pathological forms of tau and αS in surviving DA neurons from DIV 7 midbrain cultures exposed to 10 and 15 µM of CLD for 5 days. The immunosignal produced with the AT8 antibody that detects abnormally phosphorylated Ser 202 and Thr 205 residues on the tau protein [49] was increased in a significant proportion of DA neurons (somas + neurites) remaining in the cultures after CLD exposure (Figure 3a,b). Figure 3b shows an increase in p-tau expression in the soma and proximal neurites of a TH^+^ neuron in a midbrain culture exposed for 5 consecutive days to 10 µM of CLD. Note that the AT8 immunosignal was also increased in some of the nondopaminergic neurons (Figure 3b). In control cultures, the AT8 immunosignal was weak or absent from TH^+^ neurons and other neurons.

In parallel, we assessed pathological p-αS expression in DA neurons surviving a 5-day exposure to 10 or 15 µM of CLD using an antibody that identifies pathogenic αS phosphorylated at serine residue 129 [36]. Unlike p-tau, p-αS was not increased in DA cell somas after CLD treatment (Figure 3c). The efficacy of our p-αS antibody in detecting the presence of p-αS aggregates (p-αSa) was not questionable, as positive inclusions were detectable in DIV 7 midbrain cultures exposed to 7 µg/mL of αS fibril (αSf) seeds for the next 5 days. Figure 3d shows the absence of a detectable p-αS immunosignal in an individual TH^+^ neuron from a midbrain culture exposed for 5 consecutive days to 10 µM of CLD. In contrast, a robust p-αS immunosignal was observed in the soma and neurites of a TH^+^ neuron in a sister culture exposed for 5 days to αSf seeds. Approximately 13% of TH^+^ cell somas contained large αSa under such treatment conditions.

### 3.4. CLD Does Not Evoke an Inflammatory Response in Glial Cells

**Figure 4:**
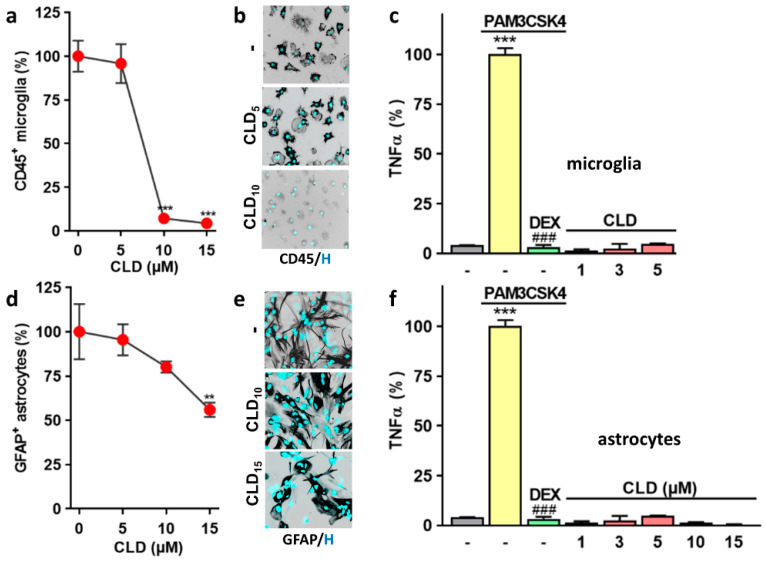
Response of glial cells to CLD intoxication. (**a**) Survival of cultured CD45^+^ microglial cells exposed to CLD (5–15 µM) for 24 h. Data are means ± SEMs (*n* = 6). *** *p* < 0.01 vs. control cultures. One-way ANOVA followed by Dunnett’s test. (**b**) Illustration showing the impact of 24 h of treatment with 5 and 10 µM of CLD on CD45^+^ microglial cells. (**c**) TNFα secretion in cultured microglial cells exposed for 24 h to CLD (1–5 µM) or the reference inflammogen PAMCSK4 (0.1 µg/mL) in the presence or absence of the immunosuppressive drug DEX (2.5 µM). Data are means ± SEMs (*n* = 6). *** *p* < 0.01 vs. untreated control cultures. ^###^ *p* < 0.001 vs. PAM3CSK4-treated cultures. One-way ANOVA followed by SNK test. (**d**) Survival of cultured GFAP^+^ astrocytes exposed to CLD (5–15 µM) for 24 h. Data are means ± SEMs (*n* = 6). ** *p* < 0.01 vs. control cultures. One-way ANOVA followed by Dunnett’s test. (**e**) Illustration showing the impact of 24 h of treatment with 10 and 15 µM of CLD on cultured GFAP^+^ astrocytes. (**f**) TNFα secretion in cultured astrocytes exposed for 24 h to CLD (1–15 µM) or to the reference inflammogen PAMCSK4 (0.1 µg/mL) with or without the immunosuppressive drug DEX (2.5 µM). Data are means ± SEMs (*n* = 6). *** *p* < 0.01 vs. untreated control cultures. ^###^ *p* < 0.001 vs. PAM3CSK4-treated cultures. One-way ANOVA followed by SNK test. In (**b**,**e**), the nuclei are counterstained with Hoechst 33,342 (H; blue).

Next, we evaluated the toxic and inflammatory potential of CLD using microglial and astrocyte cultures maintained in ACM, i.e., the culture medium used for midbrain neuronal cultures. We found that a 1-day exposure to CLD was sufficient to produce a massive loss of CD45^+^ microglial cells in a range of concentrations between 10 and 15 µM, i.e., concentrations that are also harmful for DA neurons but after longer periods of incubation (Figure 4a,b). CLD, however, had no significant impact on microglial cells when used at 5 µM. When challenged with subtoxic concentrations of CLD (≤5 µM), microglial cells failed to release TNF-α, which signifies that CLD exposure did not elicit a proinflammatory response in these cells (Figure 4c). Under the same experimental conditions, the TLR2 agonist PAM3CSK4 (0.1 µg/mL) stimulated TNF-α release through a mechanism inhibitable by 2.5 µM of the anti-inflammatory drug dexamethasone.

CLD was globally much less toxic to GFAP^+^ astrocytes than microglial cells in the same experimental setting (Figure 4d,e). Upon CLD (1–15 µM) exposure, astrocytes failed to release TNF-α, suggesting that CLD did not have the capacity to promote astrocyte activation (Figure 4f). As expected, astrocytes responded to a challenge with 0.1 µg/mL of PAM3CSK4 by releasing TNF-α and this effect was prevented by dexamethasone.

### 3.5. CLD Is Toxic and Alters the Lifespan of Live Nematodes

**Figure 5:**
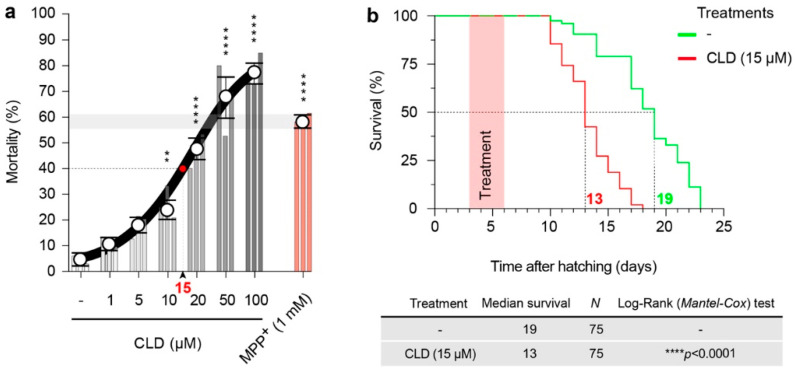
General toxicity of CLD toward *C. elegans* worms. (**a**) Synchronized young adult worms (control^GFP^) were chronically treated with CLD at various concentrations (0, 1, 5, 10, 20, 50 and 100 µM) for 3 days at 20 °C. The proportion of live worms was assessed using a stereomicroscope to manually quantify the worms in good health that responded to a mechanical stimulus. Data are means ± SEM (*n* = 50 worms per condition and per experiment with three independent experiments). ** *p* < 0.01 and **** *p* < 0.001 vs. untreated worms. One-way ANOVA followed by Dunnett’s test. A working concentration of 15 µM, inducing a mortality of approximately 40%, was most commonly used for the neurotoxicity studies. In comparison, a concentration of 1 mM of MPP^+^ used to model DA cell loss in *C. elegans* worms produced a mortality rate of 60% under the present conditions. (**b**) Lifespan assays were carried out with L1 larvae-stage transgenic worms cultured at 20 °C on NGM Petri dishes containing *E. coli* OP50, and CLD treatment was performed between day 3 and day 6 after the beginning of the assay. Upper panel: Kaplan-Meier curves showing the longevity of worms treated transiently or not treated with CLD (15 µM) for 3 days as described before and then maintained under control conditions until the indicated time. Median survival times are indicated for groups treated (red) or not treated (green) with CLD. **Lower panel**: Table indicating the median survival time for each experimental condition with statistical analysis. Data are means ± SEM (*n* = 25 worms per experiment with three independent experiments performed by an investigator blind to the treatment conditions).

Then, we defined the experimental conditions to assess CLD toxicity in an in vivo model, the nematode *C. elegans*. Accordingly, we investigated the effects of chronic exposure to increasing CLD concentrations (0, 1, 5, 10, 50 and 100 µM) on the survival of young adult worms, at 20 °C. After 72 h of exposure to CLD, the treated worms exhibited a concentration-dependent mortality that gradually increased from 10 to 100 µM (Figure 5a). For subsequent neurotoxicity experiments, a concentration of 15 µM of CLD inducing a mortality rate of approximately 40%, was most commonly used. In comparison, a concentration of 1 mM of MPP^+^ used to model DA cell loss in *C. elegans* worms [50] produced a mortality rate of 60% under the present conditions. Note that quantitative data for subsequent neurotoxicity studies were obtained in the worms that remained alive and well-formed after exposure to CLD. 

We also observed a significant decrease in the lifespan of the worms that survived to the 3-day exposure to 15 µM of CLD (Figure 5b). These observations suggest that a temporary exposure to CLD in young adult worms is sufficient to induce long-term deleterious effects on health detectable from day 10 after hatching. Based on that, we explored the mechanisms of CLD neurotoxicity in live worms that were less than 10 days old.

### 3.6. Chronic Exposure to CLD Promotes DA Cell Loss in *C. elegans* Worms

**Figure 6:**
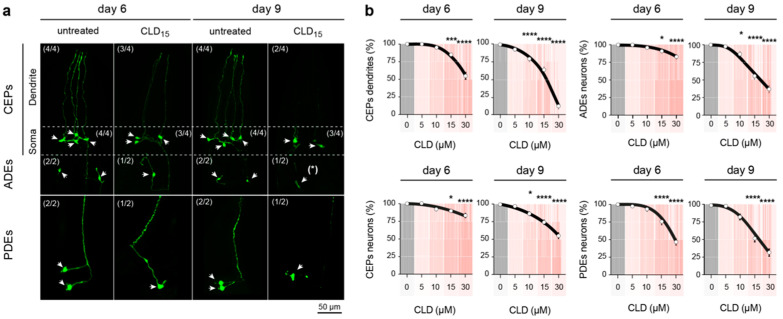
CLD-induced neurodegeneration of DA neurons in *C. elegans* worms. (**a**) Synchronized young adult *C. elegans* expressing the GFP reporter protein in DA neurons (control^GFP^) were incubated with or without CLD for 3 (day 6) or 6 days (day 9), and living worms were recovered, fixed and mounted for analysis by fluorescence microscopy (x63 objective). Representative images showing the impact of treatment with 15 µM of CLD on the three classes of DA neurons (white arrowheads) located in the head region (CEPs, *n* = 4 and ADEs, *n* = 2) and in the tail region (PDEs, *n* = 2). (**b**) Quantification of the number of CEP, ADE and PDE DA neurons and CEP dendrites after exposure to various concentrations of CLD (5, 10, 15 and 30 µM). Data are means ± SEMs (*n* = 50 worms per experiment with three independent experiments performed by an investigator blind to the treatment conditions). * *p* < 0.05, *** *p* < 0.001, **** *p* < 0.01 vs. untreated worms. One-way ANOVA followed by Dunnett’s test.

Next, we addressed the neurotoxicity of CLD for DA neurons in the nematode *C. elegans*. Precisely, we used a transgenic line of *C. elegans* expressing the GFP reporter in the dopaminergic system, which, in this animal, comprises two pairs of cephalic (CEP) neurons and one pair of anterior deirid (ADE) neurons in the head and one pair of posterior deirid (PDE) neurons in a posterior lateral position [27,39]. Specifically, we exposed synchronized young adult worms (3 days after hatching) to various CLD concentrations (5, 10, 15, 30 µM) for 3 or 6 days. Taking advantage of the transparency of *C. elegans*, we were able to monitor the neurodegenerative changes affecting DA neurons without an immunocytochemical procedure (Figure 6a).

In the *C. elegans* control^GFP^ animals exposed to 5 µM of CLD, we did not detect any significant lesion of the dopaminergic system, irrespective of the exposure time. When the worms were exposed to 10 µM of CLD, a limited but significant loss of CEP, ADE and PDE DA neurons was observable, but only after an exposure time of 6 days. When the concentration of CLD was raised to 15 µM, a 3-day exposure was sufficient to promote neuronal loss within the three populations of DA neurons. This loss was further amplified if the exposure to 15 µM of CLD was extended by 3 days. Specifically, we estimated that under these conditions, CEP, ADE and PDE DA neurons were reduced by approximately 25%, 45% and 50%, respectively (Figure 6a,b). DA cell loss was further aggravated in worms exposed to 30 µM of CLD for either 3 or 6 days.

In the CLD-treated control^GFP^ worms, we also comparatively estimated the loss of DA cell bodies and dendrites in the population of CEP DA neurons (Figure 6a,b). We established that the loss of dendrites was always more severe than the loss of cell bodies in this population of DA neurons, suggesting that deficits in dopaminergic function precede the loss of DA cell bodies in *C. elegans* animals. In particular, we found that 48% of CEP DA cell somas and 90% of the corresponding dendrites were lost in nematodes exposed to 15 µM of CLD for 6 days (Figure 6a,b). Based on these data, a concentration of 15 µM of CLD was used in the subsequent experiments with *C. elegans*.

### 3.7. *C. elegans* Worms Exposed to CLD Exhibit Locomotor Behavior Deficits Due to Alterations in Food Perception

To assess dopaminergic dysfunction in the *C. elegans* worms treated with CLD, we exposed various lines of *C. elegans* for 3 days to 15 µM of CLD and measured their capacity to slow down when encountering food (bacteria). Indeed, this locomotor behavior, known as the BSR, is mediated by dopaminergic CEP, ADE and PDE neurons, which have sensory endings in the cuticle that detect the presence of bacteria by a tactile response [45,51,52].

**Figure 7:**
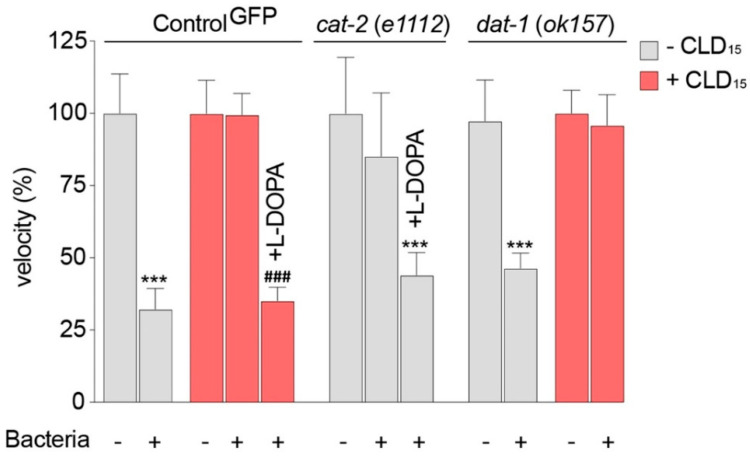
Basal slowing response of *C. elegans* worms exposed to CLD. Synchronized young adult worms from the control^GFP^ and *dat*−*1* (*ok157*) mutant strains were exposed for 3 days to 15 µM of CLD and then placed on a solid medium supplemented or not with food (bacteria) to evaluate the changes in velocity between these two conditions. When specified, L-DOPA (1 mM) was also added to the plates 3 h before assessment. Velocity was also evaluated in *cat*−*2 (e1112)* mutant animals exposed or not exposed to L-DOPA (1 mM). Data values are expressed as means ± SEMs (*n* = 30 worms per condition and experiment, with three independent experiments performed by an investigator blind to the experimental conditions). *** *p* < 0.001, vs. corresponding line, off food. ^###^ *p* < 0.001, vs. the corresponding line, off food treated with CLD. One-way ANOVA followed by Dunnett’s test.

In the control^GFP^ line, we observed that exposure to 15 µM of CLD compromised the BSR as the animals did not slow down when encountering bacteria (Figure 7). A normal slowing behavior was, however, restored in the worms exposed to CLD pretreated with 1 mM of L-DOPA. As expected, L-DOPA also restored a slowing behavior in *cat-2* (*e1112*) mutant worms deficient in TH and consequently deprived of DA. This further validates that our BSR assay specifically detects changes in dopaminergic behaviors under the present experimental conditions.

Finally, we also tested the impact of CLD in the *dat-1* (*ok157*) line, which lacks the DA transporter, as this transport system could possibly facilitate CLD accumulation in DA neurons, as it also does with other dopaminergic toxins, such as 6-OHDA and MPP^+^ [39,47]. This was not the case here, however, as the BSR remained compromised in the *dat-1* (*ok157*) mutant worms exposed to 15 µM of CLD.

### 3.8. CLD Exposure Promotes Abnormal Tau Phosphorylation in a Transgenic *C. elegans* Line Expressing a Human 1N4R Tau Isoform

**Figure 8:**
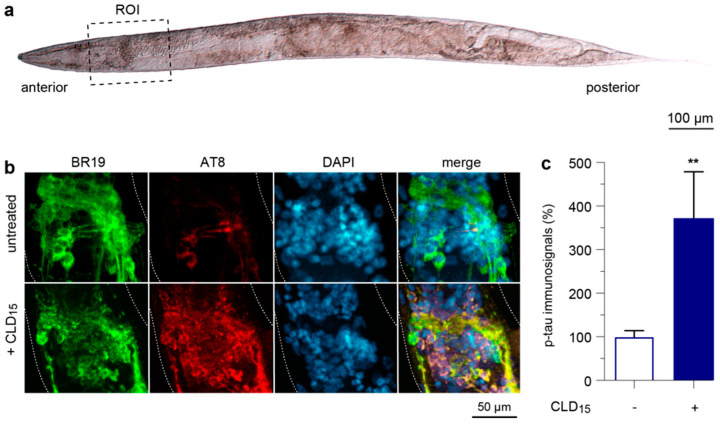
Chronic CLD exposure promotes p-tau expression in transgenic *C. elegans* worms. (**a**) Young adult *C. elegans* worm imaged with *Nomarski* optics. The black dashed rectangle defines the region of interest (ROI). (**b**) Synchronized young adult worms were treated or not treated with CLD for 3 days, and the living worms were recovered for fixation before being processed for the immunodetection of total tau (green) or p-tau (red) using BR19 and AT8 antibodies, respectively. Samples were then mounted for fluorescence imaging by confocal microscopy (x63 objective). Note that the immunosignal of p-tau (but not total tau) was dramatically increased by CLD within the anterior nerve ring. (**c**) Quantitative analysis of the p-tau immunosignal within the ROI. Data are means ± SEM (*n* = 50 worms per experiment, with three independent experiments performed by an investigator blind to the experimental conditions). ** *p* < 0.01 vs. untreated worms. Unpaired *t*-test.

To test the possibility that CLD exposure could also promote abnormal tau phosphorylation in intoxicated animals, we used a transgenic *C. elegans* line that panneuronally expresses a human wild-type 1N4R tau isoform [40]. Specifically, synchronized worms carrying the tau transgene were incubated with 15 µM of CLD for 3 days and then processed for the immunodetection of total tau and abnormally phosphorylated tau using BR19 and AT8 antibodies, respectively (Table 1) [53,54]. We more specifically analyzed the tau immunosignals in neuronal cells located within the anterior nerve ring and associated ganglia (Figure 8a). As expected, a visual examination of the immunostained *C. elegans* samples showed that the tau transgene was robustly expressed in the control and CLD-treated conditions (Figure 8b). While the p-tau immunosignal in neurons from the untreated worms was low, it was robustly elevated in CLD-treated *C. elegans*. A quantitative assessment demonstrated that the p-tau immunosignal was increased approximately 3.5-fold in the neurons from the anterior nerve ring in the CLD-treated worms (Figure 8c).

### 3.9. CLD Exposure Also Affects Cholinergic and Serotoninergic Neurotransmitter Systems in *C. elegans* Worms

**Figure 9:**
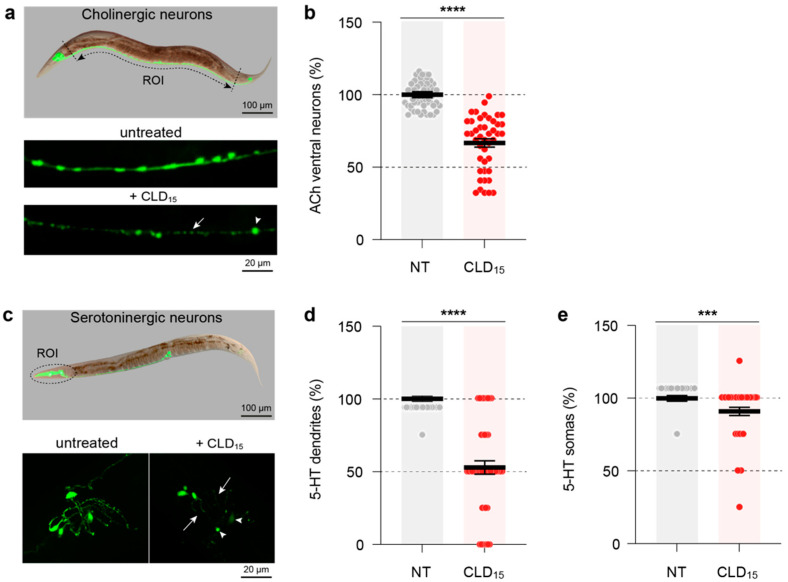
*C. elegans* worms chronically exposed to CLD develop lesions in nondopaminergic neurotransmitter systems. Synchronized young adult worms expressing the GFP reporter protein in cholinergic or serotoninergic neurons were exposed for 3 days to 15 µM of CLD, and living worms were recovered for fixation before being processed for imaging by fluorescence microscopy. (**a**) **Upper panel**: Whole-body Nomarski/fluorescence image of a young adult *C. elegans* showing the entire cholinergic neurotransmitter system of the worm. **Lower panel**: Fluorescence images showing cholinergic neurons from the median ventral cord of *C. elegans* worms treated or not with CLD. Note the substantial loss of cholinergic cell bodies (white arrowhead points to a surviving neuron) within the same segment of the ventral cord (white arrow). (**b**) Number of ventral cord cholinergic neurons in *C. elegans* worms treated or not treated with 15 µM of CLD. (**c**) **Upper panel**: Whole-body Nomarski/fluorescence image of a young adult *C. elegans* showing the entire serotoninergic neurotransmitter system of the worm. The ROI points to head serotoninergic neurons. **Lower panel**: High magnification fluorescence images showing serotoninergic neurons (white arrowheads) in the head of *C. elegans* worms treated or not treated with 15 µM of CLD. Note that there is a substantial loss of serotoninergic dendrites (white arrows) in the ROI. (**d**) Number of serotoninergic dendrites in the head of *C. elegans* worms treated or not with 15 µM of CLD. (**e**) Number of serotoninergic neurons in the head of *C. elegans* worms treated or not treated with 15 µM of CLD. Data are means ± SEM (*n* = 50 worms per experiment with three independent experiments performed by an investigator blind to the treatment conditions). *** *p* < 0.001 and **** *p* < 0.0001 vs. untreated worms. Unpaired *t*-test.

To assess whether other neurotransmitter systems might be affected by CLD exposure, we used *C. elegans* transgenic lines expressing the GFP reporter protein in either cholinergic (LX929) or serotoninergic (SK4013) systems [55,56]. Synchronized young adult worms of both genotypes were exposed for 3 days to 15 µM of CLD before being processed for morphological analyses. In the LX929 transgenic line exposed to 15 µM of CLD, we observed a significant loss of cholinergic motor neurons located within the ventral nerve cord (Figure 9a). The cell counting of a median segment of the ventral nerve cord revealed that approximately 33.2% of the cholinergic cell somas were lost in this area in the CLD-treated worms (Figure 9b). In the transgenic SK4013 line, we evaluated neurodegenerative changes affecting the population of serotoninergic neurons located in the head (Figure 9c). After CLD exposure, the loss of head serotoninergic cell bodies was relatively limited, as, on average, it did not exceed 9%; however, 47% of these somas had lost their serotoninergic dendrites (Figure 9d,e).

## 4. Discussion

Using cell culture systems of mouse midbrain DA neurons and brain glial cells together with a *C. elegans* in vivo model, we showed that the pesticide CLD has the capacity to promote the death of DA neurons and other populations of brain neurons, but not to promote glial neuroinflammation. CLD-mediated neurodegeneration appears to occur as a direct consequence of oxidative stress-mediated insults and mitochondrial disturbances. Remarkably, CLD also showed the capacity to promote phosphorylation of the aggregation-prone protein tau (but not of α-synuclein) both in midbrain cultured neurons and in a transgenic *C. elegans* strain expressing a human form of tau in neurons, indicating that CLD has the potential to contribute to atypical forms of degenerative Parkinsonism where DA cell degeneration is associated with a predominant tau pathology. 

### 4.1. Neurotoxic Effects of CLD on Midbrain-Cultured DA Neurons

CLD exerted potent toxic effects on DA neurons in midbrain cultures. The neurotoxic effects were closely dependent on the concentrations of CLD applied to the cultures and on the duration of exposure to this compound. The concentration of CLD that killed 50% of DA neurons after a 5-day exposure was estimated at 7.9 µM. Interestingly, concentrations of this order were also reported to reduce Ca^2+^ and K^+^ channel currents and cause the dysfunction of the Na/K ATPase in catecholaminergic PC12 cells [57]. Whether these activity defects also contribute to CLD-induced DA cell death in our model system, however, remains to be established.

As expected, we found that the uptake of DA—an index of the survival and function of DA neurons—was also severely impaired following CLD exposure. Similar to what we already observed with MPP^+^, the active metabolite of the prototypical dopaminergic toxin MPTP [47], we found that CLD (10, 15 µM) caused a decrease in DA uptake that was proportionally much more pronounced than the loss of TH^+^ neurons. This suggests that DA neurons that remain visually detectable after treatment with these concentrations of CLD are in fact already largely dysfunctional. Accordingly, the remaining TH^+^ neurons exhibited shrunken cell bodies and dystrophic neurites. However, the decrease in DA uptake and the loss of TH^+^ cell numbers were comparable in midbrain cultures exposed to lower concentrations of CLD, producing limited toxic effects on DA neurons. This suggests that after exposure to subtoxic concentrations of CLD, the remaining TH^+^ neurons were still functional and not irreversibly affected by the treatment.

Aging being considered a primary risk factor for PD and atypical forms of Parkinsonism [58], we wished to determine whether the state of maturation of midbrain cultures could modify the vulnerability of DA neurons to CLD. Contrary to our expectation, older DA neurons exposed to CLD did not demonstrate a higher vulnerability to degeneration, indicating that the neurotoxic potential of CLD may depend predominantly on pesticide exposure duration and concentrations. Note, however, that we do not know to what extent the increased maturation of the cultures reflects biological aging.

### 4.2. DA Neurons from the Nematode *C. elegans* Are Also Highly Vulnerable to CLD

The midbrain cell culture system used for this study may present some intrinsic limitations, as it may not accurately model all aspects of brain neurodegeneration. In particular, our toxicity assay does not take into consideration the fact that CLD might be partly degraded by enzymatic systems in vivo and might be distributed in specific areas of the brain after systemic exposure [8,59]. To move toward a more integrated model system, we used a line of *C. elegans* expressing GFP under the DAT-1 promoter in DA neurons [27]. This line offers the advantage of allowing the visualization of DA neurons without performing TH immunodetection.

We found that in *C. elegans* worms, CLD exerts severe toxic effects on DA neurons at a concentration of 15 µM, which is also quite effective in midbrain cultures. Remarkably similar to our observations in vitro, DA cell loss developed progressively as a function of time between 3 and 6 days of continuous exposure to CLD. Among the three groups of DA neurons distributed in the head (CEP, ADE) and body (PDE) [39], ADE and PDE neurons appeared most vulnerable to CLD. We also noted that the dendrites of CEP and PDE neurons were generally severely affected by CLD exposure, suggesting that, similar to what we observed in midbrain cultures, DA neurons from CLD-treated worms become dysfunctional before ultimately dying. Based on these different observations, we may assume that CLD comes into contact with DA neurons in the head and the body after being absorbed through the mouth and/or the cuticle. This is not surprising in view of the high lipophilicity of this compound, denoted by a log *p* value of ~5.41 [60]. 

Interestingly, damage to the dopaminergic system was further confirmed behaviorally by showing that the BSR is impaired in CLD-treated worms transferred onto a bacterial lawn [45,61]. A similar response was observed in *cat-2* (*e1112*) mutants [62], i.e., TH-null mutant worms that were not treated with CLD, indicating that the defective behavior of CLD-treated worms was a direct consequence of DA cell degeneration. Confirming this interpretation, deficits in BSR observed in the CLD-treated worms or in *cat-2 (e1112)* mutants were reversed by treatment with L-DOPA, the necessary precursor to DA.

### 4.3. The Neurotoxicity of CLD Is Not Restricted to DA Neurons

For a number of neurotoxins, such as MPP^+^, 6-OHDA and the pesticide/herbicide cocktail Paraquat/Maneb, which have the potential to kill midbrain DA neurons in animal models of PD, the DA transporter acts as an entryway [63]. The possibility that CLD could become toxic after accumulation in DA neurons through the DA transporter is, however, unlikely for the following reasons: (i) inhibiting this transport system with GBR12909 did not reduce the toxicity of CLD for DA neurons in culture, and (ii) the deficit in the slowing response observed in CLD-treated worms was also detectable in *dat-1 (ok157) C. elegans*, a deficient mutant lacking the presynaptic DA transporter [64]. We can, therefore, assume that due to its lipophilic character [60], CLD enters DA neurons by passively diffusing through the plasma membrane. This probably also explains why CLD toxicity develops only progressively over time and why this pesticide affects non-DA neurons in midbrain cultures (not shown). Similarly, nondopaminergic neuronal populations were also affected in CLD-treated *C. elegans* worms. In particular, we found that serotoninergic and cholinergic neurons underwent neurodegeneration after CLD exposure. The impact of CLD on these neurons seemed, however, to be proportionally less than that on DA neurons.

### 4.4. Impact of CLD on Brain Glial Cells

The present data indicate that CLD can exert direct effects on specific populations of neuronal cells. However, we also tested the possibility that CLD might become neurotoxic by activating microglial cells, brain parenchymal macrophages [65] or astrocytes, another type of specialized glial cell in the brain [66]. We established that the survival of cultured microglial cells was affected at concentrations of CLD that were also toxic to DA neurons. Cultured astrocytes, however, appeared much less vulnerable to CLD than microglial glial cells, which is consistent with data showing that astroglial cells are generally much more resistant to a large variety of noxious stimuli [67,68].

When placed in the presence of subtoxic amounts of CLD, neither microglial cells nor astrocytes responded by releasing TNF-α, a prototypical cytokine involved in the progression of a number of brain inflammatory states related to neurodegeneration [69], including PD [70,71]. The release of TNFα, however, was evident after exposure to the Toll-like receptor 2 agonist PAM3CSK4, used in the present setting as a reference inflammogen in both model systems [34]. Overall, this set of data clearly indicates that CLD has no intrinsic proinflammatory properties toward brain glial cells.

### 4.5. p-tau but Not p-αS Accumulates in Vulnerable DA Neurons

We wished to determine whether CLD-induced neurodegeneration could promote the formation of p-Ser-129 αS, the pathological form of αS that accumulates in Lewy bodies in PD brains [36,72], or induce tau pathological changes [73] characteristic of atypical forms of Guadeloupean Parkinsonism [17]. We found that a fraction of DA neurons in midbrain cultures overexpressed tau phosphorylated at Ser202/Thr205 in their soma and neuritic network in the course of CLD intoxication. P-tau was also strongly expressed in some of the non-DA neurons in CLD-treated midbrain cultures. Most interestingly, we also established that pathological tau phosphorylation is prominent in neurons from the anterior nerve ring in tau transgenic *C elegans* worms [74] exposed to CLD, confirming the view that CLD has the capacity to cause tau abnormalities in an in vivo setting. Conversely, p-Ser-129 αS inclusions were never seen in either dopaminergic or nondopaminergic neuronal cell bodies in the course of CLD-induced neurodegeneration. However, p-αS aggregates were readily detectable under experimental conditions known to promote αS aggregation [23], confirming our capacity to detect pathological p-αS aggregates in the present setting. Note that the possibility that CLD could also exert neurotoxic effects by worsening an already ongoing αS pathology, as previously suggested regarding Dieldrin, another organochloride pesticide [75], has not been addressed here, and should not be totally excluded.

Overall, we demonstrate that CLD promotes tau (but not αS) phosphorylation in specific subsets of neurons both in midbrain cultures and in the transgenic line of *C. elegans* worms expressing human tau. CLD is therefore more likely to promote atypical forms of neurodegenerative Parkinsonism characterized by tau abnormalities rather than PD, classified as a synucleinopathy disorder. Unexpectedly, however, dopaminergic deficits in *C elegans* worms intoxicated with CLD were well corrected by L-DOPA, which is somehow different to what is observed in atypical Parkinsonian patients for whom L-DOPA is poorly effective due to the widespread loss of postsynaptic dopaminergic receptors [15,17,58]. 

### 4.6. CLD Intoxication Results in Mitochondrial Depolarization and ROS Production

Using the fluorogenic probe DHR-123 as a tool to detect intracellular oxidative stress [22,76], we found that ROS are produced relatively early in the course of CLD-induced neurodegeneration in midbrain cultures. Precisely, ROS production was detectable after 2 days of exposure to 15 µM of CLD, a stage at which DA neurons start to degenerate. At this time, ROS were increased in a substantial fraction of midbrain neurons, presumably those becoming irreversibly committed to degeneration. Upon CLD exposure, ROS production correlated with a drop in ΔΨm, as demonstrated with the mitoprobe TMRM [77], suggesting that oxidative stress originated initially from depolarized mitochondria. This is consistent with the fact that DHR-123 is known to be preferentially retained by these organelles after oxidative conversion into its fluorescent derivative rhodamine 123 [78]. The fact that the TMRM signal was specifically decreased in the subpopulation of ROS-producing neurons indicates that CLD-mediated oxidative stress may follow ΔΨm disruption. Note, however, that ROS were not significantly enhanced after disrupting ΔΨm with the uncoupler of mitochondrial oxidative phosphorylation, FCCP, suggesting that ROS production may in fact precede mitochondrial dysfunction. Consistent with this view, an acute challenge with the oxidizing agent H_2_O_2_, sufficient to promote widespread intracellular oxidative stress, resulted in a severe drop in ΔΨm.

ROS having the potential to promote p-tau expression in other experimental settings [79,80], one may, therefore, assume that oxidative stress induced by CLD possibly contributed to p-tau induction in both midbrain cultures and tau transgenic worms. Severe oxidative stress is also believed to favor αS aggregation in some animal models [81], and it remains to be explained why CLD intoxication did not result in the formation of p-αS inclusions.

### 4.7. Potential Modulators of CLD-Mediated DA Cell Death

Given the capacity of CLD to stimulate ROS, we tested whether prototypical antioxidants known to be protective in other model systems against oxidative stress-mediated insults [22] could protect DA neurons from CLD-mediated neurodegeneration. Neither the inhibitor of lipid peroxidation Trolox-C, nor the iron chelator desferioxamine, provided substantial protection against CLD. Blocking NMDA glutamate receptors with MK-801 was similarly ineffective, suggesting that ROS produced by CLD exposure were not the consequence of glutamate-mediated excitotoxic insults [46,82]. Likewise, stimulating the glycolytic flux with 50 mM of glucose to possibly overcome ATP deficits caused by dysfunctional mitochondria [18] was also ineffective. The lack of efficacy of such treatments suggested that other key cellular functions may be concurrently impaired during CLD intoxication, leading at some point to catastrophic neurodegenerative events.

### 4.8. Circumstances under Which CLD Could Reach Neurotoxic Levels in the Human Brain

While there is no doubt that CLD possesses intrinsic neurotoxic properties, an important question is to determine under what circumstances the pesticide could possibly reach neurotoxic levels in the human brain. We can rely for that on clinical data from Cannon and collaborators reporting average and maximal blood concentrations of about 2.5 and 7 ppm of CLD, respectively (i.e., 2.5–7 µg/mL) in chemical workers developing severe neurological symptoms after chronic poisoning with the pesticide [83]. Such values are very similar to the EC50s reported to be toxic for cultured neurons in this study (7.5–10 µM, i.e., 3.7–4.9 µg/mL). Given that CLD has a good ability to cross the blood–brain barrier [8,60,84], we might assume that extracellular concentrations close to the levels leading to neurodegeneration were present in the brain of intoxicated chemical workers. Blood concentrations of CLD in the general Caribbean population were found, however, at least 50-fold lower than the levels causing neurological symptoms [83,85]; Emeville and colleagues reported highest CLD plasma values of 0.05 µg/mL in their study population [85]. Yet, we cannot totally rule out the possibility that neurotoxicity might develop at much lower concentrations of CLD than those reported here, in cases of persistent environmental exposure. Besides, a possible potentiation of CLD neurotoxicity by other pesticide contaminants present in the Caribbean ecosystems [85] should not be excluded.

## 5. Conclusions

Overall, although we are well aware of the limitations of the experimental models used for this study, our results clearly indicate for the first time that the risk of developing neurodegenerative Parkinsonism might be increased in Caribbean populations experiencing low-level but persistent environmental exposure to CLD. Our results will almost certainly further raise the awareness of the individuals most at risk of CLD exposure, leading to better compliance with protective measures against contamination. From an etiological perspective, our results may also indirectly improve our understanding of how environmental factors contribute to the development of PD and related disorders.

## Figures and Tables

**Table 1:** List and details of primary antibodies.

Primary Antibodies	Host	Working Dilution	Source	Identifier
anti-TH [LNC1]	Mouse	1:2500	Immunostar	22941
anti-TH	Chicken	1:1000	Abcam	ab76442
anti p-S202/pT205 Tau [AT8]	Mouse	1:500	Thermo Fisher Sci.	#MN1020
anti-Tau [EP2456Y] *	Rabbit	1:200	Abcam	#ab76128
anti-p-S129 αS [EP1536Y]	Rabbit	1:2500	Abcam	#ab51253
BB515 anti-CD45	Rat	1:100	BD BioSciences	#564590
anti-GFAP	Chicken	1:1000	Sigma-Aldrich	#AB5541

* Used only for *C. elegans* studies.

## Data Availability

Datasets generated during the current study are available from the corresponding author on a reasonable request.

## Supplementary Materials

The following supporting information can be downloaded at: https://www.mdpi.com/article/10.3390/cells12091336/s1, Figure S1: Impact of culture age on CLD-induced neurotoxicity in midbrain DA neurons; Figure S2: Evaluation of compounds with the potential to limit CLD neurotoxicity for DA neurons.
