# Supplementary material for: The Pesticide Chlordecone Promotes Parkinsonism-like Neurodegeneration with Tau Lesions in Midbrain Cultures and C. elegans Worms
Source: Cells. 2023 May 7;12(9):1336. doi: 10.3390/cells12091336 (PMC10177284; doi:10.3390/cells12091336)
Supplement: Supplementary file 1 [file cells-12-01336-s001.zip › cells-2301752-supplementary.pdf]

Supplementary results (Figures S1, S2)

To assess a possible impact of culture age on chlordecone (CLD)-induced neurotoxicity, we exposed DIV 14 (instead of DIV 7) midbrain cultures to 1-20 μM CLD for 5 consecutive days before fixation and processing for immunoanalysis. Similar to observations made in younger midbrain cultures, more mature dopamine (DA) neurons were killed in a concentration-dependent manner after 5 days of chronic treatment with CLD. The EC_{50} for DA cell loss was estimated at approximately 10 μM, a concentration close to that found in younger cultures (Figure S1).

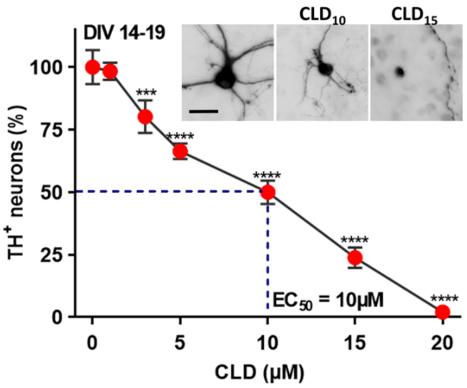

Figure S1. Impact of culture age on CLD-induced neurotoxicity in midbrain DA neurons. Survival of DA neurons as a function of increasing CLD concentration (1-20 μM) in 2-week midbrain cultures treated with this compound between DIV 14-19 and then assessed for TH immunocytochemistry. Data are means ± SEMs (n = 6). *** $p < 0.001$, **** $p < 0.0001$ *vs* untreated controls. One-way ANOVA followed by Dunnett's test. *Inset*: Representative illustrations showing the morphology of TH^+ neurons in midbrain cultures exposed or not exposed to CLD (10 or 15 μM) between DIV 14-19. Scale bar: 20 μm.

Next, we tested whether treatments capable to potentially curtail CLD-mediated insults were capable of rescuing CLD-treated DA neurons. We found that the inhibitor of lipid peroxidation, Trolox-C (10 μM), the iron chelator desferrioxamine (10 μM) and the glutamate NMDA receptor blocker MK-801 (2 μM) could not protect DA neurons from a 5-day exposure to 10 μM CLD (Figure S2). Increasing the amount of glucose in the culture medium by 50 mM was similarly ineffective.

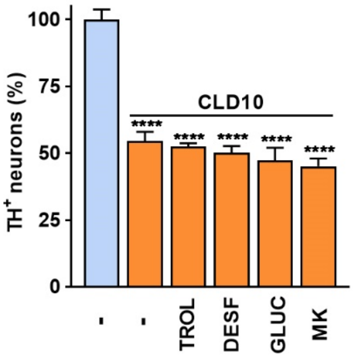

Figure S2. Evaluation of compounds with the potential to limit CLD neurotoxicity for DA neurons. Midbrain cultures treated with 10 μM CLD between DIV 7-12 in the presence or absence of the inhibitor of lipid peroxidation Trolox-C (TROL; 10 μM), the iron chelator desferrioxamine (DESF; 10 μM), the NMDA receptor antagonist MK-801 (2 μM) or high glucose (GLUC; +50 mM). Data are means ± SEM (n =3).*** $p < 0.001$ *vs* untreated control cultures. One-way ANOVA followed by Dunnett's test.
